# Associations between socioeconomic status, home food availability, parental role-modeling, and children’s fruit and vegetable consumption: a mediation analysis

**DOI:** 10.1186/s12889-023-15879-2

**Published:** 2023-05-31

**Authors:** Nithya Serasinghe, Henna Vepsäläinen, Reetta Lehto, Anna M. Abdollahi, Maijaliisa Erkkola, Eva Roos, Carola Ray

**Affiliations:** 1 Folkhälsan Research Center, Topeliuksenkatu 20, Helsinki, 00250 Finland; 2 Department of Food and Nutrition, University of Helsinki, P.O. Box 66, Helsinki, FI-00014 Finland; 3 Department of Food Studies, Nutrition and Dietetics, Uppsala University, Uppsala, Sweden; 4 Department of Public Health, University of Helsinki, P.O. Box 63, Helsinki, FI-00014 Finland

**Keywords:** Socioeconomic position, Home food environment, Physical food environment, Social food environment, Dietary quality, Healthy diet

## Abstract

**Background:**

Recent literature has suggested that associations and interactions between family socioeconomic status (SES) and home food environment influence children’s diet, but little is known about the mediation roles of parental role-modeling and food availability in the socioeconomic inequalities of children’s diet. This study aimed to determine the associations between family SES and children’s fruit and vegetable (FV) consumption and to assess the mediation roles of parental role-modeling and food availability in the above associations.

**Methods:**

Cross-sectional data of 574 Finnish children (aged 3 to 6) were analyzed. Parents completed an FFQ assessing their children’s FV consumption frequency and a questionnaire assessing SES and home food environment. Two exposure variables: parental educational level (“low”, “middle”, and “high”) and the relative family income tertiles of the family were used. The frequencies of parental role-modeling of FV and sugary food and drink (SFD) consumption, and the availability of FV and SFD at home were calculated. Single- and multiple-mediator models were created using IBM SPSS 27.0.

**Results:**

The positive association between high parental educational level and children’s FV consumption (direct effect coefficient: 2.76, 95% CI: 0.51–4.86) was partially mediated by more frequent parental role-modeling of FV consumption (indirect effect coefficient: 0.89, 95% CI: 0.10–1.76), higher availability of FV (indirect effect coefficient: 1.00, 95% CI: 0.35–1.77), and lower availability of SFD (indirect effect coefficient: -0.30, 95% CI: -0.72 – -0.01). The relative family income was not directly associated with the outcome. However, the higher relative family income level indirectly predicted the Children’s FV consumption (full mediation) through more frequent parental role-modeling of FV consumption (indirect effect coefficient: 0.91, 95% CI: 0.06–1.83) and higher availability of FV (indirect effect coefficient: 0.98, 95% CI: 0.40–1.67). Parental role-modeling on SFD consumption did not mediate any of the above associations.

**Conclusions:**

Parental educational level showed more associations with children’s FV consumption than relative family income. Our findings suggest that reducing the availability of SFD is as important as increasing the availability of FV to enhance children’s FV consumption. Future interventions to improve children’s dietary behaviors should pay greater attention to the lower SES segments of society. Longitudinal studies and intervention studies supporting these findings are needed for making meaningful recommendations for health promotion.

## Background

Adequate consumption of fruits and vegetables (FV) is considered a key component of a healthy diet [1]. The positive influence of higher family socioeconomic status (SES) on children’s FV consumption is well-documented [2–5]. Educational level and income are two major indicators of SES [6]. Previous studies conducted in high-income countries have revealed associations between high SES levels and healthy dietary intake among children [7–9]. Understanding how SES is linked to children’s diets is important for tackling such SES inequalities. The home food environment, which can be defined as any opportunity to obtain food at home [10], including social aspects such as parental role-modeling, and physical aspects such as food availability [11] may be important links between SES and children’s diets. Available evidence suggests that parental educational level may influence home food environment via nutritional knowledge and food-related parenting practices. [12]. Low-income households are less likely to purchase healthy food that are comparatively rich in fiber and low in salt, added sugar, and saturated fats [13–15] leading to a home food environment, which does not support healthy eating habits.

A role model is a significant figure or character that influences motivation and goals and inspires the behaviors of another individual [16]. Parents are identified as strong role models for children [17]. Recent studies show a clear dietary resemblance between parents and children [18, 19]. According to the available evidence, parents who consume the daily recommended amount of FV are more likely to have children who also consume the recommended daily amount of FV [20, 21]. Furthermore, the inability of parents to meet nutritional recommendations can impede healthy dietary behaviors among their children [22]. Home food availability, which refers to the presence of food at home [23], plays an important role in children’s diets [24–26]. Home availability of FV has usually been positively associated with children’s consumption of FV [27–30]. In addition, reducing the availability of unhealthy food including sugary food and drinks (SFD) at home has been effective in improving children’s diet quality [31] and healthy food consumption, including FV [32].

The concept of “mediation” can simply be defined as the transference of the effect of an independent/ predictor variable on a dependent/ outcome variable through a third variable called a mediator variable [33]. Mediation analysis can be used to study intermediate variables, such as social or physical home food environments, which may act between the independent (SES) and outcome (diet) variables [34]. Previous mediation studies of children’s diets have largely focused on psychosocial variables, such as parents’ self-efficacy, attitudes, and knowledge [35] and a limited number of studies have assessed the mediation roles of the home food environment. In addition, most previous mediation studies have focused on older children. A recent systematic review has identified home food availability and parental role-modeling of food consumption as mediators of the socioeconomic inequalities of diets among youth in European countries [36]. However, little is known about the above-mentioned mediation roles in the diets of younger children. Children of preschool age spend more time at home and are more exposed to the home food environment than older children [35]. Moreover, the dietary behaviors that are continued throughout life are developed during early childhood [35]. Therefore, knowledge of the influencing factors of diet at preschool age is important for health promotion and intervention planning. The aim of this study was to reduce the above-mentioned knowledge gap by finding evidence on possible pathways by which the aspects of the home food environment influence the diet of preschool-aged children.

**Fig. 1:**
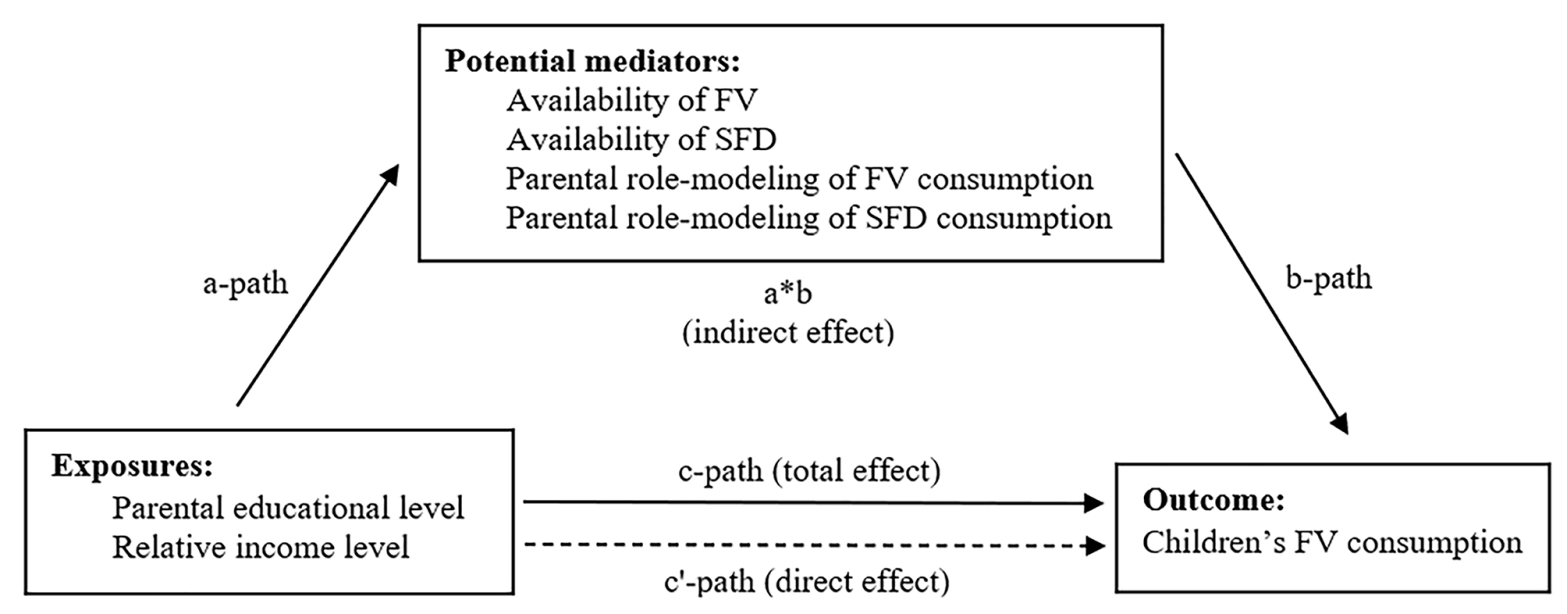
Availability of FV, availability of SFD, parental role-modeling of FV consumption, parental role-modeling of SFD consumption as mediators of the association between family SES and children’s FV consumption. a-path: association between exposure and potential mediator; b-path: association between potential mediator and outcome; c-path: overall association between exposure and outcome; c'-path: direct (unmediated) association between exposure and outcome.

In this study, we hypothesized that the parental educational level and the relative family income are positively associated with children’s consumption of FV. We also hypothesized that the above associations are mediated by home food availability (FV and SFD) and parental role-modeling of FV and SFD consumption, as illustrated in Fig. 1. To the best of our knowledge, this is the first study to include both the social and physical aspects of the home food environment with regard to preschool children’s diet simultaneously in a mediation model.

## Methodology

### Study design and participants

The DAGIS (Increased Health and Wellbeing in Preschools) project (www.dagis.fi) was aimed to promote healthy energy balance-related behaviors among preschool children in Finland. This study utilized cross-sectional data from the baseline assessment of the DAGIS intervention, a randomized controlled trial conducted in 2017–2018 [37]. Participants were recruited in two municipalities in Southern Finland: Salo and Riihimäki. All the public preschools in Salo (n = 29) and three public preschools in Riihimäki agreed to participate. Altogether, 1702 eligible children in the 3–6-year age group from 32 preschools and their families were invited to take part in the study, and 802 (47% of invitees) consented. Baseline data were collected from September to October 2017. During the data collection, the parents of 728 children completed the parental questionnaire. The University of Helsinki Ethical Review Board of the Humanities and Social and Behavioral Sciences approved the DAGIS intervention study as ethically acceptable in May 2017 (22/2017).

### Measurements and variables

#### Exposure variables: parental educational level and relative family income

Two SES indicators: education and income, were used as the exposures of this study. The parental questionnaire had six response categories for the educational level of the parents: (1) comprehensive school, (2) vocational school, (3) high school, (4) bachelor’s degree or polytechnic degree, (5) master’s degree, and (6) licentiate/doctorate. When applicable, the parent who filled in the questionnaire reported the highest educational level for both themselves and the other parent living in the same household. The answers were classified into three educational levels: “low” (Categories 1, 2, and 3), “middle” (Category 4), and “high” (Categories 5 and 6).

The parent reported the average net income of the entire household per month using 10 predefined answer options (1 = Less than EUR 500; 2 = EUR 500–999; 3 = EUR 1000–1499; 4 = EUR 1500–1999; 5 = EUR 2000–2499; 6 = EUR 2500–2999; 7 = EUR 3000–4999; 8 = EUR 5000–7499; 9 = EUR 7500–10 000; 10 = over EUR 10 000). All regular income, such as capital gains, pensions, and other social benefits, including child benefits, were considered household income. The answers were restructured by assigning an exact value as 1 = EUR 500, 2 = EUR 750, 3 = EUR 1250, 4 = EUR 1750, 5 = EUR 2250, 6 = EUR 2750, 7 = EUR 4000, 8 = EUR 6250, 9 = EUR 8750, and 10 = EUR 10 000. A standard equivalence scale [38] was used to weigh the net income of the household by the number of family members and to calculate the relative family income [39]. This scale weighted the household’s net income as the first adult in the household = 1.0, the second adult in the household = 0.5, and each child under 18 years = 0.3. The relative family income variable was then categorized into tertiles. The relative family income in the “low”, “middle”, and “high” levels was less than EUR 1668, EUR 1668–1905, and more than EUR 1905, respectively.

#### Outcome variable: children’s FV consumption

A Food Frequency Questionnaire (FFQ) consisting of 51 items was used to assess children’s FV and SFD consumption outside preschool hours during the preceding week. The parents reported the number of times during the preceding week that their child had consumed each food or drink item outside preschool. An electronic version of the FFQ was emailed to the parent. Upon request, printed copies of the FFQ were provided and collected in sealed envelopes. Three response options were given for each food item in the FFQ: “not at all”, “times per day”, and “times per week”. Parents were instructed to tick the “not at all” column or write the number in one of the other columns. Four food items (fresh fruits, fresh berries, fresh vegetables, and cooked or canned vegetables) were extracted from the FFQ for this study. The consumption frequencies were summed up to compute the outcome variable. Two outcome variables were also computed by summing up the consumption frequencies of fruits and vegetables separately. However, we only report the findings for the combined outcome of FV consumption because splitting up fruit and vegetable consumption in the outcome did not change the results (data not shown). The FFQ was a modified version of a 47-item paper version of the same FFQ that has shown fairly good validity in ranking FV consumption compared to food record data [40]. The FV items have also shown moderate reproducibility [41].

### Potential mediators

#### Food availability

A previously developed and published tool for assessing food availability[25, 42] was modified and used to collect food availability data [27]. Modifications were made by adding commonly consumed food and drink items and removing the food items considered unnecessary in the Finnish context [23]. The questionnaire had four food items under the FV group and fourteen food and drink items under the SFD group (see Table 1). The parents who answered the questionnaire reported the availability of food/drink items at home as never, rarely, sometimes, often, and always. Values from 1 to 5 were allocated to each answer (1 = never, 5 = always). The values for food items in each food group were summed up and averaged to compute the mediator variables; availability of FV and availability of SFD.

#### Parental role-modeling

The questions assessing parental role-modeling were adapted from a previously validated tool [43]. The question, “During the past week, how often did you consume (food group) when your child was around?” was repeated for all five food groups: vegetables, fruits, sugary everyday foods, sugary treats, and sugary drinks (see Table 1). Six response options were allocated for each question: 1 = not once, 2 = 1–2 times, 3 = 3–4 times, 4 = 5–6 times, 5 = daily, and 6 = more than once a day. The above responses were converted into 0, 1.5, 3.5, 5.5, 7, and 10.5 respectively. The values for food items in each food group (see Table 1) were summed up to compute the two mediator variables: parental role-modeling of FV consumption (times/week) and parental role-modeling of SFD consumption (times/week).

**Table 1:** Food items included in outcome and mediator variables

Food group		Food items in the FFQ
Fruits and vegetables	Fruits	Fresh fruits
		Berries (fresh, frozen)
	Vegetables	Fresh vegetables
		Cooked and canned vegetables
Sugary food and drinks	Sugary everyday foods	Flavored yoghurt and quark
		Puddings
		Berry, fruit, and chocolate porridge
		Sugar-sweetened cereals and muesli
		Berry and fruit stews
	Sugary treats	Ice cream
		Chocolate
		Sweets
		Cakes and pastries^a^
		Sweet cookies and cereal bars
	Sugary drinks	Sugar-sweetened soft drinks
		Flavored and sweetened milk- and plant-based drinks
		Sugar-sweetened juice
		Sugar-sweetened juice

^a^ Cakes, cupcakes, buns, pies, and sweet pastries

### Covariates

The children’s age and gender were included in the mediation analysis as covariates. The parents reported their children’s gender and birth date. Age was calculated according to the date of birth and was used as a continuous variable.

### Statistical analysis

The Statistical Package for Social Sciences (SPSS) version 27.0 [44] was used for the descriptive and statistical analysis. The FV consumption variable was assessed for normal distribution, and outliers of at least three standard deviations from the mean (3SD) were removed from the dataset (N = 15). Outcome and mediator variables were checked for multicollinearity using linear regression. As the variance inflation factors for all the combinations among the outcomes and mediators were below 5, we assumed that no multicollinearity was present in the model [45]. We assessed the correlations among the variables using the Spearman correlation test.

The mediation analysis of this study was conducted in two stages. Firstly, in the single-mediator model, we checked the individual mediation effects of the four potential mediators (availability of FV, availability of SFD, parental role-modeling of FV, and parental role-modeling of SFD) on the association between the exposures (parental educational level and relative family income) and the outcome (children’s FV consumption). The significance of the total effect, direct effect, a-path and b-path effects (see Fig. 1) were determined on the basis of the p-value. The indirect effect (a*b), which was the mediational effect in which the exposure influenced the outcome through a certain mediator, was considered statistically significant if its confidence interval did not contain zero. Secondly, in the multiple-mediator model, the significant mediators identified from the single-mediator model were tested simultaneously. We also checked the independent mediation effect of each mediator and the total effect of the model. Mediation analyses were conducted using the PROCESS Version 3 Macro for SPSS [46]. The “low” educational group and “low” relative family income group were treated as the reference categories.

In addition to the above-mentioned analysis, we split our original outcome (FV) into two variables: fruit consumption and vegetable consumption and conducted the analysis again separately for the two outcomes (data not shown). As we observed no notable differences between the results before and after splitting the outcome variable, we only present and discuss the results for one outcome: children’s FV consumption.

## Results

### Descriptive statistics

Of the 698 parents who filled out the parental questionnaire, 91% were mothers. The children from whom we had data on at least one of the exposures, one of the mediators, and the outcome were eligible for the analysis (N = 574). The children’s mean FV consumption frequency was 21 times per week (Table 2). Parents reported role-modeling of FV and SFD consumption a mean of nine (ranging from 2 to 12) and five (ranging from 3 to 14) times per week in front of the child, respectively, outside preschool time.

**Table 2:** Descriptive characteristics of participants and variables

Characteristic/ variable	N (missing)	Mean ± SD or percentage	Minimum - Maximum
Age (years)	574 (0)	5.2 ± 1.03	2.92–6.67
Gender	574 (0)		
Boy	316	55%	
Girl	258	45%	
Parental educational level^a^			
Low	166	29%	
Middle	267	47%	
High	140	24%	
Total	573 (1)		
Relative income^b^			
Low	146	28%	
Middle	203	39%	
High	176	33%	
Total	525 (49)		
Availability of FV^c,h^	570 (4)	4 ± 0.6	2–5
Availability of SFD^d,h^	565 (9)	2 ± 0.4	1–3
Parental role modeling of FV consumption (times per week)^e,i^	570 (4)	9 ± 2.5	2–12
Parental role modeling of SFD consumption (times per week)^f,i^	572 (2)	5 ± 1.8	3–14
Children’s FV consumption (times per week)^g,^	574 (0)	21 ± 10.8	0–57

FV = fruits and vegetables, SFD = sugary food and drinks. ^a^ Categories of parental educational level; ‘low’ = comprehensive school, vocational school, secondary school, ‘middle’ = polytechnic school or bachelor’s degree, ‘high’ = master’s degree or licentiate/doctoral degree. ^b^ Cut-offs for relative income tertiles; less than EUR 1666.67 per person = ‘low’, EUR 1666.67 < > 1904.76 per person = ‘middle’, higher than EUR 1904.76 per person = ‘high’. ^c^ Availability of FV = average household availability of four FV items in FFQ. ^d^ Availability of SFD = average household availability of thirteen SFD items in FFQ. ^e^ Parental role-modeling of FV consumption = weekly frequency of parents eating FV in presence of child. ^f^ Parental role-modeling of SFD consumption = weekly frequency of parents eating SFD in presence of child. ^g^ FV consumption of children = weekly consumption frequency of four FV items in FFQ. ^h^ values from 1 to 5 were allocated to each food (1 = never, 5 = always). ^i^ values from 1 to 11 were allocated to each food (1 = not even once, 11 = more than once a day)

### Correlation analysis

Higher parental educational level was weakly associated with children’s increased FV consumption (r = 0.13), availability of FV (r = 0.13) and parental role-modeling of FV (r = 0.08). Higher relative family income was weakly associated with increased availability of FV (r = 0.16), increased availability of SFD (r = 0.12), and more frequent parental role-modeling of FV (r = 0.10). Table 3 shows the correlations between all the variables.

**Table 3:** Spearman correlation coefficients between variables (n = 503–578)

	1.	2.	3.	4.	5.	6.
Exposure variables						
1. Parental educational level						
2. Relative income of family	0.33^**^					
Outcome variable						
3. Children’s FV consumption	0.13^**^	0.08				
Potential mediators						
4. Availability of FV	0.13^**^	0.16^**^	0.32^**^			
5. Availability of SFD	0.07	0.13^**^	-0.08	0.17^**^		
6. Parental role-modeling of FV consumption	0.09^*^	0.09^*^	0.43^**^	0.30^**^	-0.08	
7. Parental role-modeling of SFD consumption	0.05	-0.04	-0.02	-0.07	0.35^**^	0.12^**^

^*^Correlation is significant at 0.05 level

^**^ Correlation is significant at 0.01 level

### Single-mediator model

Parental educational level showed a direct effect on the children’s FV consumption under all four potential mediators. According to the a-path coefficients, higher parental educational level was positively associated with three potential mediators: availability of FV (b: 0.96, 95% CI: 0.38–1.54), availability of SFD (b: 1.13, 95% CI: 0.54), and parental role-modeling of FV (b: 1.40, 95% CI: 0.18–2.62) (Table 4). Further, higher availability of FV (b: 1.43, 95% CI: 1.10–1.75), greater parental role-modeling of FV (b: 0.83, 95% CI: 0.68–0.98), and lower availability of SFD (b: -0.21, 95% CI: -0.38 – -0.05) were positively associated with the children’s FV consumption (b-path). Availability of FV (b: 1.37, 95% CI: 0.51–2.30), availability of SFD (b: -0.24, 95% CI: -0.61 – -0.001), and parental role-modeling of FV (b: 0.80, 95% CI: 0.30–1.30) had indirect effects (a*b) on the association between parental educational level and children’s FV consumption. Parents with “middle” and “high” educational levels role-modeled FV consumption more frequently, which was associated with more frequent children’s FV consumption than among the “low” educational level. The households of parents in the “middle” and “high” educational levels had higher availability of FV, which in turn was associated with more frequent children’s FV consumption. Higher availability of SFD in the households of parents with “middle” educational level was associated with less frequent children’s FV consumption.

**Table 4:** Mediation effects on association between parental educational level, relative income level, and children’s FV consumption (Single-mediator models, adjusted for gender and age of child)

Exposure	Mediator	Exposure levels	Direct effect (c’)		a-path		b-path		Indirect effect (a*b)	
			b (SE)	95% CI	b (SE)	95% CI	b (SE)	95% CI	b (SE)	95% CI
Parental educational level	Availability of FV (N = 569)	Low (ref)								
		Middle	-0.41 (1.01)	-2.39–1.57	0.80 (0.26)**	0.30–1.30	1.43 (0.17)**	1.10–1.75	1.15 (0.43)^+^	0.36–2.02
		High	2.76 (1.17)*	0.46–5.06	0.96 (0.30)**	0.38–1.54			1.37 (0.46)^+^	0.51–2.30
	Parental role-modeling of FV consumption (N = 569)	Low (ref)								
		Middle	-0.42 (0.97)	-2.33–1.48	1.33 (0.54)*	0.27–2.38	0.83 (0.08)**	0.68–0.98	1.10 (0.48)^+^	0.20–2.09
		High	3.01 (1.12)**	0.80–5.22	1.40 (0.62)*	0.18–2.62			1.16 (0.51)^+^	0.18–2.20
	Availability of SFD (N = 564)	Low (ref)								
		Middle	0.95 (1.07)	-1.14–3.05	1.13 (0.54)*	0.08–2.19	-0.21 (0.08)*	-0.38 – -0.05	-0.24 (0.16)^+^	-0.61 – -0.001
		High	4.43 (1.23)**	2.01–6.84	1.13 (0.62)	-0.09–2.34			-0.24 (0.18)	-0.66–0.03
	Parental role-modeling of SFD consumption (N = 571)	Low (ref)								
		Middle	0.71 (1.06)	-1.37–2.79	-0.01 (0.33)	-0.65–0.63	-0.01 (0.14)	-0.28–0.25	0.0002 (0.05)	-0.10–0.12
		High	4.21 (1.23)**	1.79–6.63	0.06 (0.13)	-0.19–0.31			-0.002 (0.06)	-0.1–0.13
Relative income	Availability of FV (N = 521)	Low (ref)								
		Middle	-0.13 (1.12)	-2.34–2.07	0.59 (0.28)*	0.04–1.13	1.41 (0.18)**	1.06–1.76	0.83 (0.42)^+^	0.04–1.68
		High	0.88 (1.17)	-1.42–3.18	1.05 (0.29)**	0.49–1.61			1.49 (0.46)^+^	0.66–2.43
	Parental role-modeling of FV consumption (N = 523)	Low (ref)								
		Middle	-0.06 (1.07)	-2.15–2.04	0.87 (0.58)	-0.27–2.02	0.85 (0.08)**	0.70–1.01	0.75 (0.51)	-0.25–1.77
		High	1.31 (1.11)	-0.88 –3.48	1.31 (0.60)*	0.12–2.50			1.13 (0.53)^+^	0.10–2.18
	Availability of SFD (N = 518)	Low (ref)								
		Middle	0.78 (1.18)	-1.54–3.10	1.23 (0.58)*	0.09–2.37	-0.19 (0.09)*	-0.36 – -0.01	-0.23 (0.17)	-0.62–0.01
		High	2.57 (1.22)*	0.16–4.98	1.57 (0.60)**	0.39–2.75			-0.29 (0.19)	-0.75–0.01
	Parental role-modeling of SFD consumption (N = 524)	Low (ref)								
		Middle	0.70 (1.18)	-1.62–3.01	-0.78 (0.36)*	-1.48 – -0.07	0.03 (0.14)	-0.25–0.31	-0.02 (0.13)	-0.33–0.22
		High	2.45 (1.22)*	0.05–4.84	-0.71 (0.37)	-1.44–0.02			-0.02 (0.12)	-0.30–0.20

FV = fruits and vegetables, SFD = sugary food and drinks. b = unstandardized coefficient. SE = standard error. * Statistically significant effect at p-level < 0.05. ** Statistically significant effect at p-level < 0.01. ^+^ Statistically significant indirect effect at p-level < 0.05

Relative family income showed a direct effect on children’s FV consumption under two potential mediators: availability of SFD (b: 2.57, 95% CI: 0.16–4.98) and parental role-modeling of SFD consumption (b: 2.45, 95% CI: 0.05–4.84). The associations between relative family income and potential mediators (a-path) showed that a higher relative family income was associated with higher availability of FV (b: 1.05, 95% CI: 0.49–1.61), more frequent parental role-modeling of FV consumption (b: 1.31, 95% CI: 0.12–2.50), higher availability of SFD (b: 1.57, 95% CI: 0.39–2.75), and less frequent parental role-modeling of SFD consumption (b: -0.78, 95% CI: -1.48 – -0.07). Higher availability of FV (b: 1.41, 95% CI: 1.06–1.76), more frequent parental role-modeling of FV consumption (b: 0.85, 95% CI: 0.70–1.01) and lower availability of SFD (b: -0.19, 95% CI: -0.36 – -0.01) were positively associated with children’s FV consumption (b-path). “Middle” (b: 0.83, 95% CI: 0.04–1.68) and “high” (b: 0.1.49, 95% CI: 0.66–2.43) relative family income levels were associated with more frequent children’s FV consumption through higher availability of FV. In addition, “high” (b: 1.13, 95% CI: 0.10–2.18) relative family income level was positively associated with children’s FV consumption through more frequent parental role-modeling of FV consumption.

### Multiple-mediator model

Table 5 displays the simultaneous and independent mediation roles of all the significant single mediators in the multiple-mediator model. The association between parental educational level and children’s FV consumption was partially mediated by the availability of FV, parental role-modeling of FV, and availability of SFD. The association between relative family income and children’s FV consumption was fully mediated by the availability of FV and parental role-modeling of FV consumption.

**Table 5:** Mediation effects on associations between parental educational level, relative income of family, and children’s FV consumption (Multiple-mediator model, adjusted for gender and age of child)

Exposure	Multiple-mediator model	Exposure level	Total effect (c)		Direct effect (c’)		Indirect effect (a*b)	
			b (SE)^##^	95% CI	b (SE)^##^	95% CI	b (SE)^##^	95% CI
Parental educational level	*Total mediation effect (N = 557)*							
	All significant mediators in the Single-mediator model together	Low (ref)						
		Middle	0.71 (1.08)	-1.41–2.83	-0.64 (0.96)	-2.53–1.24		
		High	4.27 (1.25)**	1.872– 6.72	2.76 (1.10)*	0.51–4.86		
	Availability of FV	Low (ref)						
		Middle					0.80 (0.33)^+^	(0.22–1.50)
		High					1.00 (0.36)^+^	(0.35–1.77)
	Parental role-modeling of FV consumption	Low (ref)						
		Middle					0.84 (0.39)^+^	(0.013– 1.66)
		High					0.89 (0.42)^+^	(0.10–1.76)
	Availability of SFD	Low (ref)						
		Middle					-0.29 (0.16)^+^	(-0.66 – -0.04)
		High					-0.30 (0.18)^+^	(-0.72 – -0.01)
Relative income	*Total mediation effect (N = 519)*							
	All significant mediators in the Single-mediator model together	Low (ref)						
		Middle	0.72 (1.19)	-1.61–3.05	-0.43 (1.05)	-2.49–1.64		
		High	2.44 (1.23)*	0.03–4.85	0.55 (1.10)	-1.61–2.71		
	Availability of FV	Low (ref)						
		Middle					0.54 (0.28)^+^	0.02–1.12
		High					0.98 (0.33)^+^	0.40–1.67
	Parental role-modeling of FV consumption	Low (ref)						
		Middle					0.61 (0.43)	-0.25–1.46
		High					0.91 (0.46)^+^	0.06–1.83

FV = fruits and vegetables, SFD = sugary food and drinks. b = unstandardized coefficient. SE = standard error. * Statistically significant effect at p-level < 0.05. ** Statistically significant effect at p-level < 0.01. ^+^ Statistically significant indirect effect at p-level < 0.05

## Discussion

The present study had two aims: The first was to determine the associations between both parental educational level and relative family income and the FV consumption of preschool children in Finland. The second was to explore the mediation roles of the availability and parental role-modeling of FV and SFD. In line with our hypotheses, parental educational level was positively associated with children’s FV consumption. However, relative family income did not show a direct association with the outcome. Availability of FV, parental role-modeling of FV, and availability of SFD partially mediated the association between parental educational level and children’s FV consumption. The association between relative family income and children’s FV consumption was fully mediated by the availability of FV and parental role-modeling of FV consumption.

The positive association between parental educational level and children’s FV consumption found in this study is in line with the findings of previous studies conducted in Finland [47, 48] and in other countries of similar development levels [7, 8, 49–51]. According to a recent study, a healthy dietary pattern was more prevalent among children (aged 9 to 14) of higher-educated parents in Finland [47]. In addition, a similar analysis in Finland that used data of the same age group as this study revealed that lower parental educational level was associated with less frequent children’s FV consumption[48].

In the present study, relative family income was not directly associated with children’s FV consumption. Cross-sectional analyses with similar or compatible variables conducted in Finland [52], Australia [52–54], and the United Kingdom[52] have shown findings that are in line with ours. In addition, two cohort studies in France[55] and South Korea[56] have also made similar findings. Finland is a country with less income disparities in society, despite the different educational levels of the population [57]. This can be one reason why family income did not predict children’s consumption of FV like parental educational level did.

Our findings imply that parental educational level is a stronger predictor of children’s FV consumption than relative family income in Finland. Previous studies have assessed the associations between individual SES indicators and children’s FV consumption, but the abilities of different SES indicators to predict FV consumption have rarely been discussed. Several studies focusing on adults have identified education as a stronger SES indicator predicting food behaviors, including FV consumption, than other SES indicators such as income and profession [58–61]. High-educated people are generally considered more knowledgeable than less-educated people [62]. Therefore, knowledge of a healthy diet may be more important for increased FV consumption than income. Income does not always reflect expenditure on food. Even families with higher incomes might struggle to prioritize a healthy diet and adequate nutrition due to other expenditures. In such situations, people may not have the resources to persuade their children to taste new fruits and vegetables, and just serve the food the children prefer. As the results of our study might have been influenced by the method we used to categorize the relative family income, future studies should use more distinctive categorization methods to assess the associations of relative family income.

Parental role-modeling of FV consumption appeared to be a mediator of the associations between the two exposures (parental educational level and relative family income) and FV consumption in different stages of childhood. Cross-sectional analyses of nine-month-olds [63], of adolescents[64] and of eight-year-olds[65] and 11-year-olds[66] have identified parental role-modeling as a mediator of the association between parental educational level and children’s FV consumption. In addition, a systematic review has concluded that parental role-modeling is a consistent mediator of socioeconomic inequalities in children’s food consumption during youth [36]. However, none of the above-mentioned studies focused solely on preschool-aged children. Therefore, our findings provide evidence to support the consistency of the mediation role of parental role-modeling throughout childhood. A recent systematic review has identified maternal FV consumption as a mediator of the socio-economic inequalities in 2.5- to 7-year-old children’s FV consumption, though the review did not compare maternal and paternal FV consumption [36]. A previous study by our research group showed moderate dietary resemblance in both father-child and mother-child dyads, suggesting that both parents are important role models for the child [18]. Our study did not assess the mediation effects of mothers’ and fathers’ FV consumption separately. However, 91% of the respondents to the parental questionnaire of our sample were mothers.

Parental role-modeling can be a challenging variable due to the use of different measurement methods and potential biases. Previous studies have measured parental role-modeling using questionnaires filled out by parents about eating together with their child [64, 65], FFQs [63] or 24-hour diet recall of parents [20] to determine general parental food intake. Interestingly, many of the studies mentioned above have observed parental role-modeling plays a mediation role on the association between parental educational level and children’s food consumption, regardless of the measurement method used. Self-reported dietary data are highly susceptible to social desirability bias [67]. The questions we used to assess parental role-modeling asked about parental dietary intake in the presence of the child. Reporting food consumption in the presence of the child might be similarly or even more prone to social desirability bias than a general FFQ. Therefore, FV consumption might have been over-reported and SFD consumption under-reported.

This study identified the availability of FV as a mediator of the associations between SES indicators and FV consumption. A previous cross-sectional study using data on children aged 11 in 10 European countries including Finland has identified the availability of FV as a strong mediator of the association between parental educational level and school-aged children’s consumption of FV [68]. In addition, several studies of adolescents [69] and 11-year-old children [66] with similar or compatible variables conducted in similar contexts have also found comparable evidence. Moreover, a systematic review concluded that FV availability has a consistent mediation role in the socioeconomic inequalities of FV consumption during youth [36].

Availability of SFD showed a negative mediation effect on the association between parental educational level and children’s FV consumption. A recent study has revealed a negative association between the availability of SFD and preschool children’s consumption of FV in Finland [27] but, except for our findings, no other evidence is available on the mediation role of the availability of SFD on the socioeconomic inequalities in children’s FV consumption in Finland. Previous studies have identified strong mediation roles of the availability of SFD in socioeconomic inequalities of children’s SFD consumption [70–72], but only a few studies have assessed the mediation role of SFD availability on socioeconomic inequalities in FV consumption [53, 73] the findings are not consistent. However, a very recent multivariate analysis concluded that reducing the availability of unhealthy food at home, including SFD, more effectively improves healthy dietary behaviors among school-aged children than increasing the availability of FV [32]. A possible explanation for this could be that higher availability of SFD leads to a higher intake of SFD during and in between main meals, replacing the amount of FV consumed during main meals. On the other hand, low availability of SFD could simply be a better indicator of a greater preference for FV consumption.

Similar to other Western societies, in the Finnish food culture, fruits and vegetables are not consumed in a similar manner. Vegetables are often served during main meals whereas fruits might be consumed as snacks between main meals. To obtain a deeper understanding of the associations and mediation effects, we split our original outcome into two variables: fruit consumption and vegetable consumption, and reran the analysis for new outcomes. Nevertheless, the results from before and after splitting up the FV consumption variable were similar, suggesting that regardless of their different roles in Finnish food culture, the associations between SES and fruit and vegetable consumption and the possible mediators do not differ greatly.

This study had several strengths. Using an FFQ of known validity and reliability [40, 41] which was specifically designed and developed for the DAGIS project, preschool age group, and the Finnish context is among the main strengths. We included both healthy (FV) and unhealthy (SFD) food groups and both the social (role-modeling) and physical (food availability) aspects of the home food environment in our mediation models. Instead of using a composite score for SES, we studied the individual associations of two SES indicators. The results showed that the two SES indicators predicted FV consumption in different ways. Understanding the individual behaviors of SES indicators may be highly valuable in planning interventions to minimize socioeconomic inequalities in children’s diets. We recommend that future research studies the different SES indicators and their associations with children’s diet more comprehensively, as this may reveal crucial knowledge for intervention planning.

We should also discuss the limitations of this study. The analysis of cross-sectional data does not reveal how the associations change when children grow up. The educational level of our study sample was higher than the Finnish average [74]. Accurate data on income may be more difficult to obtain than data on education level because people are often reluctant to answer questions about income [75]. We received 728 completed parental questionnaires, which included questions on income and education. Of these questionnaires, the number with missing data on educational level and household income was 17 (3% of the study sample) and 90 (16% of the study sample), respectively. These missing values and unanswered questions might have affected and attenuated the observed associations. Respondents who do not want to reveal their income are more likely to belong to the lower SES society with less education and less income [76] making the study sample seem more educated and with higher incomes than it actually was. We still found associations when the data were selective, and we can assume that these associations would have been stronger with more complete income data. Reverse causality between the mediators and the outcome is possible, and this may have influenced our findings. For example, the higher availability of FV and more frequent parental role-modeling of FV could have been caused by the children’s higher preference of eating FV. Despite the acceptable reproducibility and validity, the FFQ was filled by parents and proxy-reported dietary data may be susceptible to over-reporting and under-reporting. The accuracy level of children’s dietary data may differ according to the respondent (mother or father), and the age and gender of the respondent who answered the questionnaire [41]. Consumption frequencies do not reveal the actual quantities of food eaten, and the quantities may vary significantly from child to child. Lastly, though the children were recruited to the study from 32 preschools, the analysis was not adjusted for clustering at the preschool level. This might have affected the results. However, we only considered the variables related to home food environment and food consumption outside the preschool hours. Therefore, the effect of not adjusting for clustering at preschool level may be fairly weak.

Findings of our study are relevant for the planning of health promotion interventions in Finland and in other counties with similar contexts. To reduce the socioeconomic inequalities in food consumption and home food environment, it is important to identify the factors and mediators, which might predispose children from low SES families to poor diet and other health behaviours leading to adverse health effects later in life. Failing to identify these factors may reduce the effectiveness of the interventions and may further enhance the existing gap between low and high SES level. Our analysis revealed mediators that are less often established in low SES families and therefore, can be effectively used in future for planning of health promotion interventions. We noticed that a direct effect remained between parental educational level and children’s FV consumption after the mediator variables were accounted for. Other aspects of the home food environment which we did not study may also act as mediators and may explain the remaining direct effect. Therefore, future studies should focus on other potential mediators of socioeconomic inequalities in children’s diets found in previous studies, such as food accessibility, rules about food consumption, individual preferences [36], using food as a reward, parental influence [73], verbal rewarding [77], permissiveness [70], and maternal feeding stress [78]. Further, the potential difference between maternal and paternal role-modeling should be assessed. Children’s diets are not limited to FV. Further analyses should be conducted on the associations with other food groups. Previous studies with similar aims have largely focused on older children. We need more studies on younger children to understand the consistency of the mediation roles of the home food environment throughout childhood. In addition, future interventions should focus on getting parents to reflect on their own behaviors that may influence the eating habits of children. Preschools and child health clinics have a high reach to the parents of young children in Finland and therefore may be important channels to improving home food environment through educating parents.

## Conclusions

Compared to relative family income, parental educational level had more associations with the FV consumption of preschool children. Higher availability of FV, more frequent parental role-modeling of FV consumption, and lower availability of SFD partially mediated the association between parental educational level and children’s FV consumption. Furthermore, the higher availability of FV and more frequent parental role-modeling of FV consumption fully mediated the association between relative family income and children’s FV consumption. Future interventions and health promotion in early childhood should aim to upgrade the home food environment by increasing FV availability, decreasing SFD availability, and supporting good parental role-modeling behaviors. Further, when considering all SES levels, greater attention should be paid to the home food environment in families with lower SES levels.

## Data Availability

The data and materials of the present study are available from the corresponding author on reasonable request.

## List of abbreviations

SES: Socioeconomic status
FV: Fruit and vegetables
SFD: Sugary food and drinks
FFQ: Food frequency questionnaire
EUR: Euro
